# ‘I hear and I forget. I see and I remember. I do and I understand.’– incorporating high-fidelity medical simulation into the undergraduate nephrology course

**DOI:** 10.1080/0886022X.2020.1847722

**Published:** 2020-11-26

**Authors:** Ewa Pawłowicz, Michelle Kulesza, Aleksandra Szymańska, Anna Masajtis-Zagajewska, Maria Bartczak, Michał Nowicki

**Affiliations:** a Department of Nephrology, Hypertension and Kidney Transplantation, Medical University of Lodz, Lodz, Poland; b Medical Simulation Center, Medical University of Lodz, Lodz, Poland

**Keywords:** Nephrology, teaching, medical simulation, undergraduate medical education

## Abstract

**Background:**

Medical simulation is a teaching method, which enables the development of clinical skills by implementing a simulation scenario in a true-to-life environment, but without exposing patients to any risk. So far, there has been no information on the use of high-fidelity simulation in undergraduate clinical nephrology teaching. Aim of this study was to analyze students' opinions and reactions to the simulation module in nephrology.

**Methods:**

The survey consisting of the Satisfaction with Simulation Experience Scale (SSES) and open-ended question concerning the overall impression of classes was conducted among 103 5^th^ year medical students, who took part in the simulation training in nephrology. SSES consisted of three parts (debriefing, reasoning, education). Statements from the open-ended question were interpreted by means of the Atlas.ti software for qualitative data analysis.

**Results:**

The overall score for simulation classes was 4.39 ± 0.69 points. Students rated debriefing, reasoning and education at 4.43 ± 0.78, 4.32 ± 0.7 and 4.39 ± 0.73 points, respectively. 87.4% and 84.5% of participants agreed that simulation developed their 'clinical reasoning' and 'decision-making' skills in nephrology, respectively.

Thematic analysis revealed that students evaluated the module as 'interesting', 'useful' and 'informative', but they found number of classes significantly insufficient. Students pointed out that due to the small emphasis placed on practical aspects in the existing curriculum e.g. routes of drug administration and conversion of doses, they could not fully benefit from simulation.

**Conclusion:**

Medical simulation is a valuable constituent of the nephrology course. Putting greater emphasis on practical aspects from the beginning of training may enable students to benefit more from simulation modules.

## Introduction

Simulation was adopted to medical education from the aviation training [1]. The first simulator was used in the field of intensive care in 1960s [2]. Since then, medical simulation keeps gaining importance in procedural and clinical training in undergraduate, postgraduate, and continuing medical education. Increasing use of simulation in the education of healthcare professionals is an effective response to the rising focus on patient safety, need for new training models and standardized educational opportunities, enabling to practice and hone skills in a controlled environment [3]. The unique feature of simulation-based medical education is learning from mistakes and error management in the true-to-life conditions. It is believed that such an approach significantly reduces the number of mistakes in real practice and provides healthcare professionals with the proper attitude to cope with errors in the most efficient way [4].

Medical simulation is most widely used in teaching of emergency medicine, interventional cardiology, anesthesiology, and surgical specialties. Nevertheless, the utilization of the simulation in internal medicine is becoming more common [1].

There are several reports on incorporating medical simulation into nephrology training programs [5–9]. However, most of them concerned the use of simulation modules in the postgraduate or continuing medical education and there are only scarce data on incorporating simulation into undergraduate clinical nephrology course [5–6].

The simulation-based, small group decision-making clinical module on dialysis was described by McKegney et al. in 1981. The authors demonstrated that application of this method turned out to be an educational success by engaging students both cognitively and affectively [5].

Nephrology-related simplistic simulation module was used in the study program of second-year pharmacy students to enhance learning about pharmacotherapeutics in electrolyte imbalances and dialysis [6]. An interesting example of use of the simulation techniques was applied by Roberts et al., who performed large group simulation sessions to ingrate basic science concepts with clinical situations in the kidney physiology program [7].

A vast majority of research in the field of simulation in nephrology consider technical procedures, that is temporal hemodialysis catheter placement [7,8] or percutaneous kidney biopsy [8], and none of the studies addressed simulation of the complex management of patients with a suspected kidney disease.

Students’ satisfaction is crucial for purposeful engagement and meaningful learning and it is important to measure its level as a variable in the overall accomplishment of the learning outcomes [9].

The aim of our study was to assess students’ opinions, satisfaction and reactions to the medical simulation module incorporated into undergraduate nephrology course.

## Materials and methods

### Simulation module description

Since summer term of the academic year 2018/2019, medical simulation module has been incorporated into the undergraduate nephrology course for the 5^th^ year students of Medical Faculty at Medical University of Lodz. The whole course of clinical nephrology consists of 6 days. Each day in the morning, the seminar on clinical cases in particular condition is conducted, followed by clinical part in the ward. On one day, instead of clinical classes in the ward, the simulation module has been introduced. The course is also comprised of the lectures in nephrology that are available for students *via* e-learning university platform.

**Figure 1.**
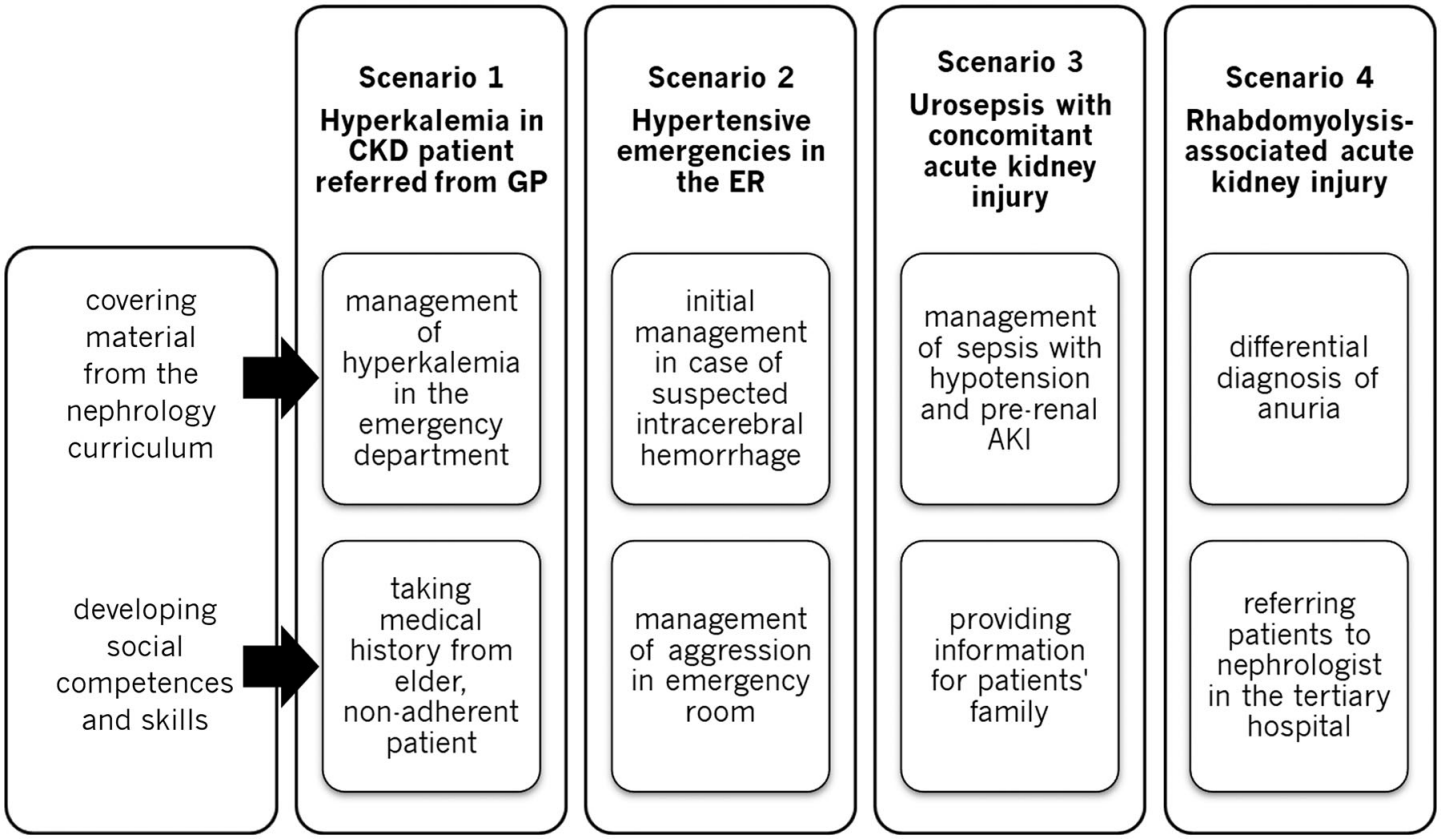
Medical simulation scenarios conducted as a part of undergraduate nephrology course by the Department of Nephrology, Hypertension and Kidney Transplantation of Medical University of Lodz.

All simulation scenarios were emergency-room based and covered four common kidney-related conditions. Besides addressing nephrological issue, each scenario enabled the development of the social competence. Students were supposed to assess the patient’s initial condition, form differential diagnosis, plan diagnostics and apply initial treatment, at the same time team-work and communication skills were trained. More detailed information on the scenarios is provided in the Figure 1. The module was performed in the high-fidelity emergency department simulation room in the Medical Simulation Center of Medical University of Lodz.

All simulation classes, being performed in groups of 5–6 students, consisted of three parts, that is (1) pre-briefing: acquainting students with the equipment of the simulation room, division of roles and tasks, presenting the introduction to the simulation scenario by the facilitator – this part lasts about 10–15 min before 1^st^ scenario, and about 5 min before subsequent scenarios; (2) simulation: proper simulation module – this part lasts 15–20 min; (3) debriefing: structured process reviewing the actions taken and its consequences enabling reflection and discussion on the performance – this part lasts about 25–30 min. One simulation class lasts about 120 min and two scenarios are conducted at that time. The module is facilitated by nephrologists, who completed the course in medical simulation provided by the local simulation center.

The classes were organized in the simulation rooms which were set up to reflect real emergency-room environment. The manikin used during the module was *HAL3201* (Gaumard, Miami, FL, USA), which is a high-fidelity simulator. Students were able to take a medical history from the manikin by means of specialized audio sound system and to perform clinical examination and assess vital signs, such as respiratory rate, heart rate, SpO_2_, etCO_2_ and noninvasive blood pressure. Breath sounds, heart sounds and bowel sounds were also available for consideration. The manikin’s initial condition and all its changes during the scenario were controlled by means of *UNI* software (Gaumard, Miami, FL, USA). Simulator was placed on real hospital bed with possibility to rise and lower the head of the bed and also to set Trendelenburg’s, reverse Trendelenburg’s and Fowler’s position. There was also another medical equipment available such as defibrillator, ventilator, vacuum suction device, infusion pumps and equipment necessary to deliver oxygen or to perform ventilation.

### Study questionnaire

Study questionnaire consisted of two parts. The first part was the Satisfaction with Simulation Experience Scale (SSES). This survey was developed as part of an ATLC (Australian Learning and Teaching Council) Project titled *Examining the impact of simulated patients and information and communication technology on nursing students' clinical reasoning* [10]. The tool was validated also in other healthcare professions’ education [11]. The survey consists of 18 items divided into three parts: (1) ‘debriefing and reflection’ addressing conditions and quality of the debriefing session; (2) ‘clinical reasoning’ concerning influence of the simulation module on decision-making and clinical reasoning skills and (3) ‘clinical learning’ referring to general clinical ability, and strengths and weaknesses of clinical performance. The participants rated the level of agreement with each statement on the 5-point Likert scale: (1) ‘strongly disagree’, (2) ‘disagree’, (3) ‘unsure’, (4) ‘agree’, (5) ‘strongly agree’.

The survey was translated into Polish by the authors of this study and distributed among 10 medical students and five faculty members in the preliminary phase of the project for checking, if items are self-explanatory. These students were not included in the study group. The feedback and remarks were applied for the final version of the survey.

Open-ended question addressed all students’ remarks and insights about the module. They were asked whether the course met their expectations and which aspect might be the most useful in the future clinical classes and upcoming medical career. It was also inquired what was the biggest challenge during this educational experience and if there were any disadvantages in terms of course content and organization. This question was a part of the study questionnaire to allow participants sharing their general perspective and address the issues that were not covered by the SSES’ statements. The information about sex and age were also gathered. The time needed to complete the questionnaire was approximately 10–15 min.

### Study group

The questionnaire was conducted in nine groups after the simulation classes were completed. Before the questionnaire was distributed, it was clearly stated by the facilitator that the participation is voluntary and the surveys are anonymous. That was also thoroughly explained in the introductory letter of the survey. The rationale and aim of the study were presented in the survey. By completing the survey, the participants gave consent to participate in the study as it was explained in the introduction of the study survey. All students agreed to answer the questionnaire. There were no exclusion criteria. One hundred and three 5^th^ year students of the medical faculty took part in the study. Basic characteristics of the study group are provided in the Table 1.

**Table 1.** General characteristics of the study population.

Characteristic	Category	N	%	Mean ± SD
Gender	male	37	35.9	
	female	58	56.3	
	missing data	8	7.8	
Total		103	100	
Age				23.9 ± 1.03

### Study design and data analysis

Data gathered from the SSES are presented as mean ± standard deviation (SD). Reliability coefficient was calculated for all SSES’ parts. Statistical analysis was performed using *Statistica* software version 13.0 PL.

For the analysis of the data gathered from the open-ended question a triangulation model was introduced. This model of qualitative research assumes a simultaneous collection of qualitative and quantitative data by pairing surveys or other quantitative measures with interviews or focus group data with subsequent integration in the final analyses [12]. The analysis of the qualitative data was performed with *Atlas.ti* version 8.0 for Windows, which is a computer-assisted qualitative data software facilitating analysis of unstructured and non-numerical data – identification of themes, patterns and meanings.

The content analysis of the students’ statements was performed separately by two raters (co-authors of this paper) followed by reconciling the consensus in terms of categories and codes. In this process 60 codes assigned to eight categories emerged. The categories were as follows: (1) simulation module – descriptions, (2) simulation module – functions, (3) simulation module – facilitation, (4) debriefing, (5) the most challenging aspect of the module, (6) shortages of current training, (7) suggestions for the future and (8) areas for improvement/critical remarks.

## Results

Results of the Satisfaction with Simulation Experience scale in the study group and percentage of participants that answered ‘strongly agree’ and ‘agree’ in the particular question are provided in Table 2. The response rate for this part of the study questionnaire was 100%.

**Table 2.** Results of the Satisfaction with Simulation Experience Scale (SSES) in the study group (*N* = 103) and percentage of participants that answered ‘strongly agree’ and ‘agree’ in the particular question.

Item	Mean ± SD	Strongly agree [%]	agree [%]
I Debriefing and reflection	4.43 ± 0.78		
The facilitator provided constructive criticism during the debriefing.	4.56 ± 0.82	70.9%	19.4%
The facilitator summarized important issues during the debriefing.	4.56 ± 0.84	71.8%	17.5%
I had the opportunity to reflect on and discuss my performance during the debriefing.	4.45 ± 0.93	64.1%	25.2%
The debriefing provided an opportunity to ask questions.	4.65 ± 0.82	79.6%	11.7%
The facilitator provided feedback that helped me to develop my clinical reasoning skills.	4.47 ± 0.96	68.9%	16.5%
Reflecting on and discussing the simulation enhanced my learning.	4.21 ± 1.00	50.5%	30.1%
The facilitator’s questions helped me to learn.	4.11 ± 1.05	43.7%	36.9%
I received feedback during the debriefing that helped me to learn.	4.28 ± 0.97	52.4%	32.0%
The facilitator made me feel comfortable and at ease during the debriefing.	4.57 ± 0.82	71.8%	18.4%
II Clinical reasoning	4.32 ± 0.70		
The simulation developed my clinical reasoning skills.	4.35 ± 0.81	51.5%	35.9%
The simulation developed my clinical decision making ability.	4.32 ± 0.84	51.5%	33.0%
The simulation enabled me to demonstrate my clinical reasoning skills.	4.22 ± 1.00	49.5%	31.1%
The simulation helped me to recognize patient deterioration early.	4.14 ± 0.86	40.8%	35.9%
This was a valuable learning experience.	4.63 ± 0.75	74.8%	17.5%
III Clinical learning	4.39 ± 0.73		
The simulation caused me to reflect on my clinical ability.	4.62 ± 0.63	68.9%	25.2%
The simulation tested my clinical ability.	4.47 ± 0.87	65.0%	22.3%
The simulation helped me to apply what I learned from the case study.	4.13 ± 1.03	46.6%	31.1%
The simulation helped me to recognize my clinical strengths and weaknesses.	4.33 ± 0.97	58.3%	26.2%
Total	4.39 ± 0.69		

Mean score in all statements was above 4.0. The best assessed part was ‘Debriefing and reflection’. The participants evaluated positively in particular safe and comfortable learning environment and the opportunity to ask questions during the debriefing session. They agreed that facilitator summarized important issues of the scenario and provided constructive criticism.

Very high mean score (4.63) was noted for the statement ‘This was a valuable learning experience’. Participants agreed also that simulation provoked them to reflect on their clinical ability.

The percentage of participants that strongly agreed or agreed with SSES’ statements exceeded 80% for all but two items. 87% and 84% of participants agreed or strongly agreed that the simulation developed their clinical reasoning skills and decision-making ability in nephrology, respectively.

To assess the internal consistency of particular parts of the survey, Cronbach alpha parameter was calculated (Table 3).

**Table 3.** Reliability coefficient (Cronbach alpha) for particular parts of Satisfaction of Simulation Experience Scale (SSES) in the study group.

Part of the SSES	Cronbach alpha
I Debriefing and reflection	0.95
II Clinical reasoning	0.86
III Clinical learning	0.84

The response rate for the open-ended question was also very high (90.3%; *n* = 93).

**Figure 2.**
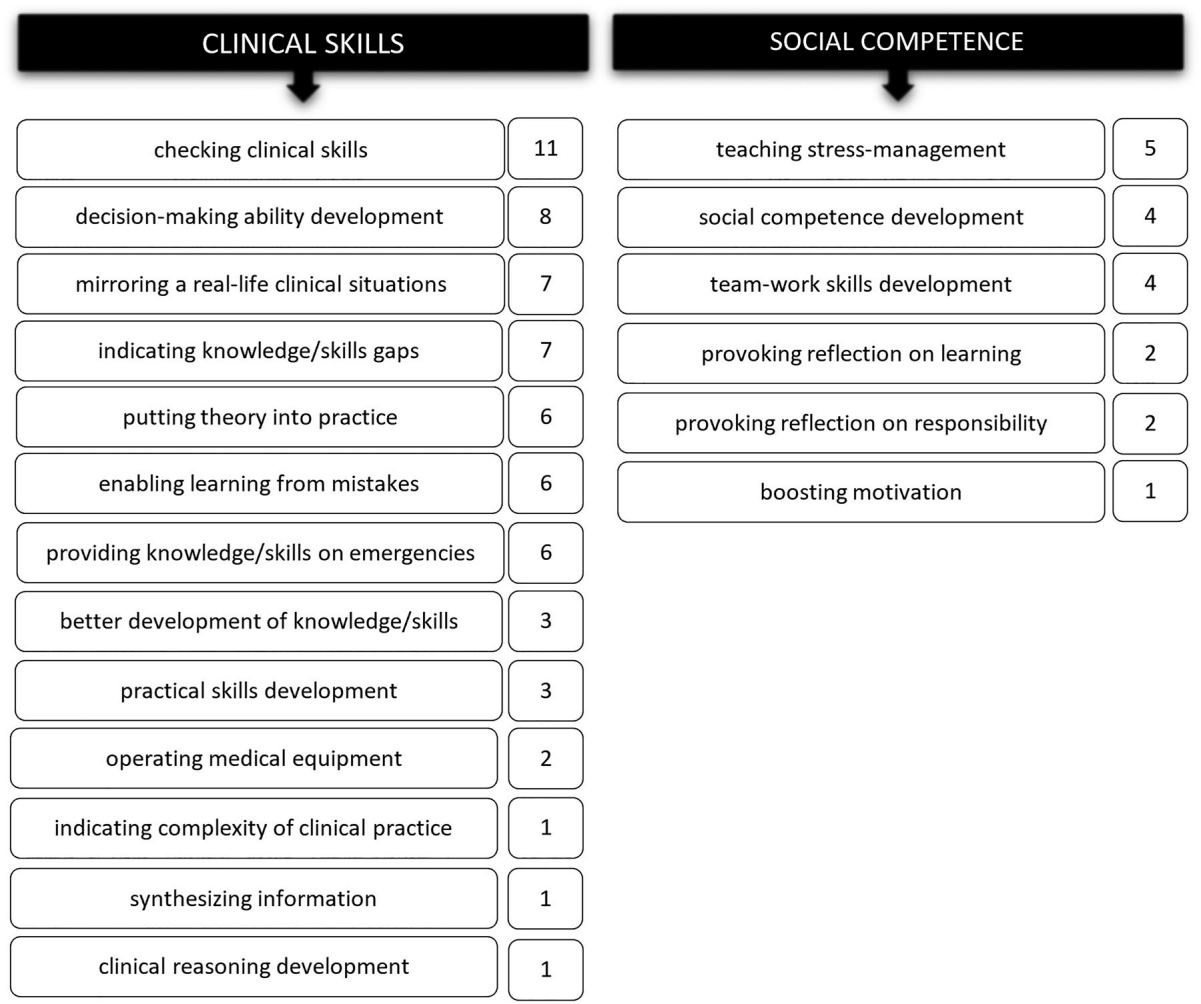
Benefits of simulation module in nephrology defined by students regarding clinical practice and social competence and the number of statements in which those codes were identified.

The participants’ responses clearly indicated their positive attitude toward the simulation module. The most commonly occurring code was *more such classes needed*. The students stated that the training met their expectations and described the simulation module as *interesting*, *valuable experience*, *very useful* and *of huge educational value*. They indicated, that simulation training is *the most valuable type of classes* from their point of view. Students defined medical simulation as beneficial at many different levels indicating its versatility (Figure 2). Students identified plenty of benefits of simulation training, however, they paid much more attention to the issues related directly to clinical practice than to social competence.

The participants valued also facilitation of the course, pronouncing comfortable learning environment, emphasis on learning from mistakes and useful guidance provided by facilitators.

Regarding the most challenging aspect of the module, students paid attention to a lack of practical knowledge on pharmacotherapy, especially drug’s doses and delivery routes (stating e.g. *We know which drugs should we give, but we don’t know how to calculate the dose and how to deliver it)*. Other challenges identified by participants included the problems with effortless use of medical equipment and team-work related issues, like cooperation and appointing the leader of the group. The students pointed out that due to the little emphasis placed on the practical aspects of clinical medicine in the existing curriculum, they cannot fully benefit from the opportunities provided by the medical simulation.

The students highlighted several deficiencies of their current training, which may be leveled out by the introduction of more simulation-based classes. These deficiencies comprised a lack of practical skills and/or contact with ‘real-life’ treatment (especially the practical aspects of pharmacotherapy) during the studies and insufficient knowledge and skills on emergencies.

The students suggested, that providing them with the list of scenarios and particular algorithms related to the simulation cases could improve the training outcomes.

As for the areas for improvement, students indicated too large training groups and some minor organizational inconveniences.

## Discussion

Nephrology as a medical specialty is in crisis. The alarming data from all around the world indicate that nephrology workforce is becoming insufficient, and expert groups on workforce planning predict a further reduction in next decades. These data seems to be especially disturbing in terms of increasing burden of CKD in the global population [13].

The causes of this phenomenon has been widely discussed. The decreasing interest in nephrology training among graduates is considered as one of the most important [15–17], followed by the general view prevailing among interns and fellows on nephrology as being unattractive and aggravating specialty.

Looking for causes of poor interest in nephrology among undergraduate trainees might be one of the keys to remediate decline of nephrology workforce. There are some limited data showing that the time spent in medical school is crucial for further career and specialty choice [14]. In the survey study about perceptions on nephrology among medical students and internal medicine residents, it was revealed that an interest in nephrology should be fostered early since medical students listed subject exposure as highly influential [15].

Parker et al. [16] indicated that students perceive adult nephrology courses as too complex and not stimulating interest and the authors hypothesized that nephrology is taught in an outdated fashion no longer stimulating medical students. Nair et al. [15] presented the strategies to improve the recruitment along the nephrology trainee continuum. At the level of undergraduate medical education two issues were identified, that is reinvigorating renal physiology courses and promoting novel educational tools. High-fidelity medical simulation in nephrology may be enumerated as such a tool. Roberts et al. [7] also concluded, that modern curriculum changes may be beneficial by promoting positive attitudes toward nephrology.

Bayefsky et al. [17] summarized the current knowledge on methods of teaching nephrology to medical students. No publications on high-fidelity clinical simulation in nephrology were referred to in that review. However, the authors invoked some studies on simulation techniques used in pulmonology and surgery curricula and presented them as the valuable examples of randomized control trials (RCTs) in medical education research.

Authors paid an attention to a lack of high-quality educational research in the field of nephrology, especially RCTs.

The Confucius’ quote that we mentioned in the title incisively indicates the benefits of medical simulation – students’ understanding of particular pathological conditions and treatment methods may be significantly improved by training clinical skills in a true-to-life environment. Such an approach is particularly valuable for domains recognized as complex and complicated, and that is a case of nephrology.

The opinions of US nephrology fellows on the use of simulation in their training were assessed in the nationwide survey by Rope et al. [18]. That study showed that there are at least preliminary data to support the use of simulation in the nephrology education. Besides, it was revealed in the report of the US Accreditation Council for Graduate Medical Education that the simulation training as part of the nephrology curricula was perceived as less prevalent than expected [19]. Introducing simulation training also at the undergraduate level of nephrology education may be therefore beneficial by increasing students’ interest with the subject and inuring them to such method of teaching, which should be widely used in the postgraduate and continuing medical education.

The interesting example of novel training approach is the NephSIM – a free, mobile-optimized, nephrology educational tool designed to teach pathophysiology with a diagnostic approach using interactive cases [20]. This intervention was so far addressed only to nephrology fellows. Even though mobile-based educational interventions preclude team-work and social skills development, such approach may be very beneficial in some extraordinary circumstances like COVID-19 pandemic global lockdown.

Our results clearly indicate a positive approach toward the medical simulation itself and the simulation module in nephrology. The benefits of medical simulation defined by our students deserve particular attention. They valued this educational method both in terms of clinical skills and social competence.

As we created the simulation module, it was our idea to focus on the emergency-room based cases, common in everyday practice, and not on specialized, complex and sophisticated conditions. Firstly, such an approach corresponds to the principle of producing graduates prepared for the general practice, and not overwhelming students with highly specialized knowledge, which they will not be able to use in the future practice. Secondly, our emergency-room based scenarios addressed complex, internal medicine cases with a special focus on kidney diseases. Such cases were appropriate and challenging enough for 5^th^ year medical students after completion of courses in internal medicine, pulmonology and cardiology.

Focusing on students’ opinions and impressions about the course might be considered among limitations of our study. Nevertheless, it is confirmed that students’ satisfaction is a key to their effective participation, which in turn may positively influence the learning outcomes of the course.

Use of the randomized, controlled research strategy in this study could result in more reliable data. However, such an approach would assume assigning only particular group of students to medical simulation training. This would be ethically questionable since we have the evidence on undeniable positive role of simulation for learning.

As for the responses in the open-ended question, the bias of priming effect must be considered. The statements provided in the SSES could influence students’ answers in that question, without their conscious guidance and intention.

Further research on the use of medical simulation in undergraduate nephrology training should comprise the assessment of the influence of this method on the learning outcomes and whether the learning was transferred into practice in the workplace [21]. The influence of the medical simulation on students’ interest in nephrology should be also addressed.

To the best of our knowledge, our report is the first study on high-fidelity, clinical simulation incorporated to the undergraduate nephrology training. Unequivocally positive perception of the simulation module is a promising beginning for further development of this teaching method in the nephrology curricula. Complexity of renal diseases and the multitude of complications that may develop in patients suffering from acute kidney injury provide unlimited possibilities for creating simulation scenarios. Our results support that putting greater emphasis on the practical aspects of training from the beginning of medical education may enable medical students to benefit more from the simulation modules.

Introducing novel teaching methods into nephrology education may lead to a raise of students’ interest in this domain and subsequently may lead to an increase of the number of graduates entering nephrology fellowship program.

## Data Availability

The data underlying this article will be shared on request to the corresponding author.

## Abbreviations

AKI: acute kidney injury
CKD: chronic kidney disease
ER: emergency room
GP: general practice
SD: standard deviation
SSES: satisfaction with simulation experience scale
